# Tissue inhibitor of metalloproteinases-1 (TIMP-1) mRNA is elevated in advanced stages of thyroid carcinoma

**DOI:** 10.1038/sj.bjc.6690198

**Published:** 1999-03

**Authors:** Y Shi, R S Parhar, M Zou, M M Hammami, M Akhtar, Z-P Lum, N R Farid, S T Al-Sedairy, M C Paterson

**Affiliations:** Department of Biological and Medical Research, King Faisal Specialist Hospital and Research Centre, PO Box 3354, Riyadh, 11211, Saudi Arabia; Department of Medicine, King Faisal Specialist Hospital and Research Centre, PO Box 3354, Riyadh, 11211, Saudi Arabia; Department of Pathology, King Faisal Specialist Hospital and Research Centre, PO Box 3354, Riyadh, 11211, Saudi Arabia; Department of Medicine, Hemel Hempstead General Hospital, Hemel Hempstead, Herts, HP2 4AD, UK

**Keywords:** TIMP-1, proteinase inhibitor, metastasis, thyroid neoplasm

## Abstract

Tumour cell invasion and metastasis is a multistep process that involves the degradation of extracellular matrix proteins by matrix metalloproteinases (MMPs). Tissue inhibitors of metalloproteinases (TIMPs) act as negative regulators of MMPs and thus prevent tumour cell invasion and metastasis by preserving extracellular matrix (ECM) integrity. In the present study we examined the expression of one member of TIMPs, TIMP-1, in 39 thyroid tumour specimens and two thyroid carcinoma cell lines (NPA and SW579). We also investigated the effect of high TIMP-1 expression on the invasive potential of NPA cells. Northern blot analysis showed that TIMP-1 mRNA levels correlated directly with tumour aggressiveness: the highest number of TIMP-1 transcripts was found in stages III and IV vs benign goitre (*P* < 0.0001). However, TIMP-1 expression was not increased in NPA and SW579 cells, both of which are derived from poorly differentiated thyroid tumours. Immunohistochemical study showed strong TIMP-1 staining in the stroma cells of advanced stages of carcinomas. Overexpression of TIMP-1 by gene transfer resulted in a significant suppression of the malignant phenotype of NPA cells as judged by an in vitro tumour invasion assay. These results suggest that high levels of TIMP-1 transcripts in advanced stages of thyroid carcinoma likely come from stroma rather than thyroid cancer cells, and TIMP-1 may function as a thyroid tumour invasion/metastasis suppressor. © 1999 Cancer Research Campaign


British Journal of Cancer (1999) 79(7/8), 1234–1239
© 1999 Cancer Research Campaign
Article no. bjoc.1998.0198

### Tissue inhibitor of metalloproteinases-1 (TIMP-1) mRNA is elevated in advanced stages of thyroid carcinoma

**Y Shi[1], RS Parhar[1], M Zou[1], MM Hammami[2], M Akhtar[3], Z-P Lum[1], NR Farid[4], ST Al-Sedairy[1] and MC Paterson[1]**

Departments of [1]Biological and Medical Research, [2]Medicine and [3]Pathology, King Faisal Specialist Hospital and Research Centre, PO Box 3354, Riyadh 11211, Saudi Arabia; [4]Department of Medicine, Hemel Hempstead General Hospital, Hillfield Road, Hemel Hempstead, Herts HP2 4AD, UK

**Summary** Tumour cell invasion and metastasis is a multistep process that involves the degradation of extracellular matrix proteins by matrix metalloproteinases (MMPs). Tissue inhibitors of metalloproteinases (TIMPs) act as negative regulators of MMPs and thus prevent tumour cell invasion and metastasis by preserving extracellular matrix (ECM) integrity. In the present study we examined the expression of one member of TIMPs, TIMP-1, in 39 thyroid tumour specimens and two thyroid carcinoma cell lines (NPA and SW579). We also investigated the effect of high TIMP-1 expression on the invasive potential of NPA cells. Northern blot analysis showed that TIMP-1 mRNA levels correlated directly with tumour aggressiveness: the highest number of TIMP-1 transcripts was found in stages III and IV vs benign goitre ($P < 0.0001$). However, TIMP-1 expression was not increased in NPA and SW579 cells, both of which are derived from poorly differentiated thyroid tumours. Immunohistochemical study showed strong TIMP-1 staining in the stroma cells of advanced stages of carcinomas. Overexpression of TIMP-1 by gene transfer resulted in a significant suppression of the malignant phenotype of NPA cells as judged by an in vitro tumour invasion assay. These results suggest that high levels of TIMP-1 transcripts in advanced stages of thyroid carcinoma likely come from stroma rather than thyroid cancer cells, and TIMP-1 may function as a thyroid tumour invasion/metastasis suppressor.

**Keywords:** TIMP-1; proteinase inhibitor; metastasis; thyroid neoplasm

Invasion and metastasis define malignancy and are major causes of cancer mortality. Tumour metastasis is a complex multistep process that involves invasion of the surrounding tissues through the confining basement membrane, penetration of blood or lymphatic vessels (intravasation), and exiting vessels at distant sites (extravasation) to form secondary tumours (Liotta and Stetler-Stevenson, 1991; Kohn and Liotta, 1995). Since extracellular matrix (ECM) and basement membrane components provide the main physical barriers against tumour cell invasion, proteolytic degradation of these structures is a critical step for invasion and metastasis (Liotta et al, 1986; Stetler-Stevenson et al, 1993). Among the enzymes involved in this degradation are the metalloproteinases (MMPs), a family of $Zn^{2+}$-dependent endopeptidases produced by host and tumour cells (Matrisian, 1990; Woessner, 1991) that include interstitial collagenase (MMP-1), gelatinase A (MMP-2, 72-kDa gelatinase or type IV collagenase), gelatinase B (MMP-9, 92-kDa gelatinase or type IV collagenase), and stromelysin (MMP-3). MMP-2 and MMP-9 are of particular importance in tumour cell invasion, because they can degrade type IV collagen, the main structural component of the basement membrane (Liotta et al, 1986). It has been shown that tumour cells expressing high levels of these enzymes are highly metastatic (Liotta et al, 1980; Nakajima et al, 1987; Murphy et al, 1989; Streenath et al, 1992; Bernhard et al, 1994).

*Received 13 October 1997*
*Revised 22 July 1998*
*Accepted 29 July 1998*

*Correspondence to:* Y Shi, MBC 3, Department of Biological and Medical Research, King Faisal Specialist Hospital and Research Centre, PO Box 3354, Riyadh 11211, Saudi Arabia

The activity of MMPs is regulated at various stages, including transcription, secretion, proenzyme activation and inhibition by tissue inhibitors of metalloproteinases (TIMPs) (Matrisian, 1990; Liotta et al, 1991; Woessner, 1991). To date, four TIMPs, encoded by four distinct genes, have been identified: TIMP-1 (Carmichael et al, 1986), TIMP-2 (DeClerck et al, 1989; Goldberg et al, 1989; Stetler-Stevenson et al, 1989), TIMP-3 (Pavloff et al, 1992), and TIMP-4 (Lew et al, 1997). Several studies have demonstrated that TIMP-1 and TIMP-2 play an important role in preventing tumour cell invasion and metastasis (Albini et al, 1991; DeClerck et al, 1991, 1992; Porto et al, 1991; Montgomery et al, 1994). Other studies have, however, shown a complex relationship between TIMPs and tumour invasiveness. Elevated TIMP-1 or TIMP-2 expression was found to be correlated with the extent of tumour cell invasion in non-Hodgkin's lymphoma (Kossakowska et al, 1991), gliomas (Nakano et al, 1995), colon (Lu et al, 1991) and breast carcinomas (Visscher et al, 1994).

It is not known whether TIMP-1 plays a role in thyroid tumour invasion and metastasis. In the present study, we therefore investigated TIMP-1 gene expression in 39 primary thyroid tumours to see whether there is a correlation between TIMP-1 expression and the aggressiveness of the disease. We also transfected human TIMP-1 cDNA into a papillary thyroid carcinoma cell line (NPA) to study the effect of TIMP-1 expression on its invasive potential using an in vitro tumour invasion assay.

### MATERIALS AND METHODS

All tumour specimens were obtained at surgery and were immediately frozen in liquid nitrogen and stored at –70°C until processed. The clinical staging of thyroid tumours was based on the TNM classification introduced in 1987 by the International Union

Against Cancer (Hermanek and Sobin, 1987). Briefly, for patients 45 years and over: stage 1 – microcarcinoma (< or = 1 cm) without lymph node involvement; stage 2 – tumours (> 1 cm) confined to the thyroid without lymph node involvement; stage 3 – any T-stage with lymph node involvement or tumours with invasion of peri-thyroidal tissue; stage 4 – distant metastasis and anaplastic tumours; for patients under 45 years: stage 1 – any T-stage with or without lymph node involvement; stage 2 – distant metastasis. Six multinodular goitres (adenomatous hyperplasia), 28 papillary carcinomas, two follicular carcinomas and three anaplastic carcinomas were studied.

The human papillary thyroid carcinoma cell line, NPA, was kindly provided by Dr James A Fagin, Division of Endocrinology and Metablism, University of Cincinnati Medical Center, Cincinnati, OH, USA, and the squamous thyroid carcinoma cell line, SW579, was obtained from ATCC (Rockville, MD, USA).

The full-length human TIMP-1 cDNA probe was obtained from thyroid RNA by reverse transcription-polymerase chain reaction (RT-PCR) using the following primers (Carmichael et al, 1986): 5′-CATCGCCGCAGATCCAGCGCCCAG-3′ (sense), 5′-GGCTTCTGCTTCCACTCCGGGC-3′ (anti-sense). The PCR product was subcloned into a TA cloning vector (pTIMP-1/TA) (Invitrogen Co., San Diego, CA, USA) and verified by DNA sequencing.

The oligonucleotide probe for 18S ribosomal RNA was synthesized and the sequence is as follows: 5′-GGTCAGCGCTCGTCG-GCATGTAATAG-3′.

### RNA extraction and Northern hybridization

Total RNA was extracted from thyroid tumour samples and cell lines by the conventional guanidinium thiocyanate–phenol–chloroform method (Chomczynski and Sacchi, 1987). Twenty micrograms of total RNA were fractionated on a 1% agarose gel containing 2.2 M formaldehyde and blotted onto a nylon membrane (Hybond-N, Amersham) by capillary transfer. The accuracy of RNA loading was monitored by ethidium bromide staining of ribosomal RNA and later by hybridization to an oligo-probe for 18S ribosomal RNA as previously described (Shi et al, 1991). The TIMP-1 cDNA probe was labelled with [$\alpha$-$^{32}$P]dCTP to a specific activity of $10^9$ cpm/μg using Pharmacia's random primer labelling kit. Hybridization was performed at 42°C for 18 h in 6 × SSPE, 10 mM EDTA, 5 × Denhardt's solution, 0.5% sodium dodecyl sulphate, 100 μg ml$^{-1}$ denatured salmon testis DNA, and 50% formamide. The membranes were then washed twice over 15 min in 2 × SSPE at 65°C and exposed to Kodak XAR-5 film at –70°C with intensifying screens.

Following autoradiography, band intensity of TIMP-1 mRNA was quantitated by a scanning densitometer (Bio-Rad model GS-670) and normalized by comparison with the intensity of the 18S ribosomal RNA band.

### Immunohistochemical procedures

Tumours were frozen and stored at –70°C prior to embedding in OCT compound. Sections (5 μm) cut from the frozen tissue blocks were fixed in cold acetone for 10 min before staining. Slides were then incubated with TIMP-1 monoclonal antibody (2 μg ml$^{-1}$ in phosphate-buffered saline) for 30 min at room temperature and stained using mouse UniTect immunoperoxidase staining kit according to the manufacturer's instructions (Oncogene Science Inc., Cambridge, MA, USA). Mouse IgG from the kit was used as negative control.

### Construction of pTIMP-1/CMV vector

The pTIMP-1/TA was digested with Hind III and Xba I restriction endonucleases to obtain TIMP-1 cDNA. The resulting fragment is oriented such that the 5′-end of the TIMP-1 cDNA is close to the Hind III site. The Hind III/Xba I fragment was then ligated into Hind III/Xba I sites of pRc/CMV, a eukaryotic expression vector from Invitrogen Co. (San Diego, CA, USA). The construct (pTIMP-1/CMV) was verified by DNA sequencing.

### Transfection assay

NPA cells were cultured in F-12 medium with 10% fetal calf serum, penicillin (100 U ml$^{-1}$), streptomycin (100 μg m$^{-1}$l), and fungizone (25 μg ml$^{-1}$) at 37°C in a humidified atmosphere containing 5% carbon dioxide. Transfection was initiated when the NPA cell culture was 70% confluent. Twenty micrograms of pTIMP-1/CMV were transfected into the cells using calcium phosphate precipitation as described previously (Chen and Okayama, 1987). Seventy-two hours after transfection, 400 μg ml$^{-1}$ G418 (Geneticin; Life Technologies Inc., Grand Island, NY, USA) were added and the cells were cultured for 3 weeks to select stable transfectants. Seven out of 200 surviving clones were characterized by Northern blot analysis and two clones were selected for use in a tumour invasion assay: TP-1 (high TIMP-1 expression), and TP-5 (low TIMP-1 expression). NPA cells transfected with the vector (pRc/CMV) alone were used as a control.

### In vitro tumour invasion assay

The invasion assay was performed to quantitate the relative degree of invasiveness of pRc/CMV.NPA (control), TP-1, and TP-5 thyroid carcinoma cells, as described previously (Parhar et al, 1995). Briefly, 3 μm pore size filters in the so-called Transwell insert (Costar, Cambridge, MA, USA) were coated with 25 μl of basement membrane matrigel (1 mg ml$^{-1}$) (collaborative research, Bedford, MA, USA) and dried under a hood. The Transwell insert was then placed in a Costar 24-well cluster plate. Sub-confluent cultures were labelled for 30 h with 1 μCi ml$^{-1}$ $^{125}$I-deoxyuridine (74 TBq mmol$^{-1}$, Amersham, Aylesbury, UK). The cells were trypsinized, washed and resuspended in complete F-12 medium. Viable cells (2.5 × $10^5$) were added to the upper compartment of the chamber in a total volume of 0.5 ml medium. The lower compartment of the chamber was filled with 0.5 ml of medium. After incubation for 96 h at 37°C in a humidified atmosphere containing 5% carbon dioxide, tumour cells that had migrated to the lower surface of the filters were recovered with trypsin–EDTA and counted in a gamma counter (1272 Clini gamma, Turku, Finland). All experiments were performed in triplicate and repeated three times.

### Statistical analysis

The significance of difference between means was analysed by the unpaired Student's *t*-test (two-tailed test). A value of $P < 0.05$ was considered significant.

© Cancer Research Campaign 1999

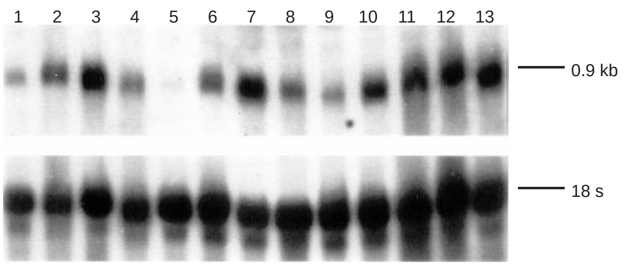

**Figure 1** Northern blot analysis of TIMP-1 mRNA levels in 13 thyroid tumour specimens. Twenty micrograms of total RNA were electrophoresed on an agarose/formaldehyde gel and blotted onto a nylon membrane. Hybridization was carried out with a full-length human TIMP-1 cDNA probe (upper panel), and an oligoprobe for 18S ribosomal RNA to monitor the actual RNA loading (lower panel). Goitre: lanes 1, 9; stage I thyroid cancer: lanes 5, 8; stage II thyroid cancer: lanes 4, 6, 7; stage III thyroid cancer: lane 3; stage IV thyroid cancer: lanes 2, 10–13. Samples 1–13 correspond to tumour specimens 1, 37, 11, 18, 35, 19, 20, 21, 2, 22, 38, 23 and 39, respectively, shown in Table 1

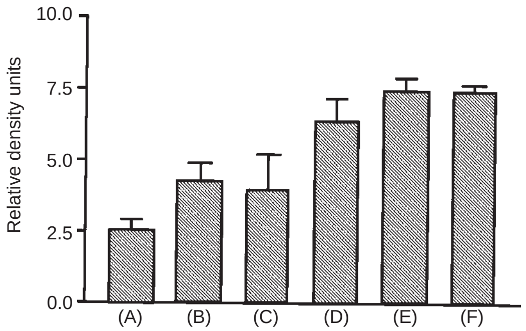

**Figure 2** Densitometric determination of the relative TIMP-1 mRNA levels. TIMP-1 transcripts were quantitated densitometrically from Northern blots and were expressed as density units relative to the signals obtained with the 18S rRNA probe. To allow comparisons among separate Northern blots, two common samples were included on each blot and TIMP-1 signals were normalized to the values obtained with these RNA samples. Values are expressed as a mean ± SEM. (**A**) Multinodular goitre ($n$ = 6); (**B–E**) differentiated thyroid cancer stage I ($n$ = 14), stage II ($n$ = 6), stage III ($n$ = 7) and stage IV ($n$ = 3), respectively, (**F**), anaplastic cancer ($n$ = 3)

**Table 1** TIMP-1 gene expression in thyroid tumours

| Tumour | Histology | Stage | Age | Sex | TIMP-1 expression[a] |
|---|---|---|---|---|---|
| 1 227889 | Multinodular Goitre | | 31 | F | 3.2 |
| 2 216951 | Multinodular Goitre | | 39 | F | 3.3 |
| 3 258127 | Multinodular Goitre | | 35 | M | 2.4 |
| 4 280061 | Multinodular Goitre | | 30 | F | 1.5 |
| 5 266427 | Multinodular Goitre | | 40 | F | 1.8 |
| 6 186282 | Multinodular Goitre | | 27 | F | 3.4 |
| 7 238533 | Papillary Ca | I | 24 | F | 3.2 |
| 8 280331 | Papillary Ca | I | 23 | F | 5.6 |
| 9 280499 | Papillary Ca | I | 26 | F | 1.9 |
| 10 284942 | Papillary Ca | I | 36 | F | 1.3 |
| 11 242132 | Papillary Ca | III | 63 | F | 8.4 |
| 12 277092 | Papillary Ca | I | 27 | F | 3.9 |
| 13 281524 | Papillary Ca | I | 36 | M | 6.0 |
| 14 282201 | Papillary Ca | I | 31 | F | 8.7 |
| 15 264860 | Papillary Ca | IV | 68 | F | 6.9 |
| 16 285061 | Papillary Ca | I | 21 | F | 4.0 |
| 17 285248 | Papillary Ca | I | 24 | F | 1.6 |
| 18 243117 | Papillary Ca | I | 35 | F | 5.5 |
| 19 150121 | Papillary Ca | III | 45 | F | 4.6 |
| 20 291156 | Papillary Ca | I | 40 | F | 8.2 |
| 21 259277 | Papillary Ca | I | 23 | F | 3.9 |
| 22 158236 | Papillary Ca | IV | 47 | F | 7.5 |
| 23 242029 | Papillary Ca | IV | 70 | F | 7.8 |
| 24 230021 | Papillary Ca | III | 59 | F | 8.8 |
| 25 260552 | Papillary Ca | I | 25 | F | 3.9 |
| 26 272560 | Papillary Ca | III | 60 | M | 6.1 |
| 27 292307 | Papillary Ca | III | 70 | F | 3.5 |
| 28 278600 | Papillary Ca | III | 46 | M | 7.4 |
| 29 294915 | Papillary Ca | I | 32 | F | 2.1 |
| 30 286226 | Papillary Ca | III | 58 | F | 6.6 |
| 31 023894 | Papillary Ca | II | 47 | F | 1.6 |
| 32 284471 | Papillary Ca | II | 41 | F | 8.8 |
| 33 296221 | Papillary Ca | II | 72 | F | 5.9 |
| 34 258028 | Papillary Ca | II | 52 | F | 3.0 |
| 35 178890 | Follicular Ca | II | 48 | F | 1.5 |
| 36 224253 | Follicular Ca | II | 56 | F | 2.9 |
| 37 287120 | Anaplastic Ca | IV | 76 | F | 6.7 |
| 38 246240 | Anaplastic Ca | IV | 81 | M | 7.6 |
| 39 219342 | Anaplastic Ca | IV | 43 | F | 8.2 |
| NPA | | | | | 3.7 |
| SW579 | | | | | 2.4 |

[a]TIPM-1 mRNA levels expressed as density units relative to signals obtained with the 18S rRNA probe. M, male; F, female; Ca, carcinoma.

### RESULTS

### TIMP-1 gene expression in thyroid tumour tissue specimens

The abundance of TIMP-1 mRNA was examined in tissues from six multinodular goitres and 33 malignant thyroid tumours. TIMP-1 was expressed in all the tissues studied. Figure 1 shows Northern blot hybridization results for a representative panel of tumour specimens. The level of TIMP-1 expression was quantitated by a densitometer and compared among tumours of different stages (Figure 2 and Table 1). Multinodular goitres have a value of 2.60 ± 0.34 (mean ± SEM); stage I differentiated thyroid carcinomas, 4.27 ± 0.62; stage II, 3.95 ± 1.18; stage III, 6.49 ± 0.73; and stage IV, 7.40 ± 0.27. Anaplastic carcinomas have a value of 7.50 ± 0.44. Therefore, TIMP-1 expression started to increase in the early stage of thyroid carcinoma (stage I) as compared with that in benign multinodular goitres ($t$ = 2.56, $P$ < 0.05) (Figure 1 and Table 1). A significant increase in TIMP-1 expression was observed in samples with advanced stages of thyroid carcinoma (stages III and IV) as compared to multinodular goitres ($t$ = 6.91, $P$ < 0.0001) or stage I tumours ($t$ = 2.75, $P$ < 0.05). We also analysed TIMP-1 expression in both NPA and SW579 cell lines which are derived from poorly differentiated thyroid carcinomas. Interestingly, TIMP-1 expression was not increased in either NPA or SW579 cells as compared to multinodular goitres (Table 1). The level of TIMP-1 expression and relevant clinical data on the patients studied are summarized in Table 1.

In order to address the question of whether tumour cells or stroma cells are the source of the increased TIMP-1 expression, we performed immunohistochemical analysis of seven papillary carcinoma specimens with increased TIMP-1 mRNA levels. All of them demonstrated strong TIMP-1 immunostaining in the stroma cells with no, or weak, tumour cell staining. A representative immunohistochemical staining is shown in Figure 3.

### Effects of TIMP-1 overexpression on the invasiveness of NPA cells

The presence of high levels of TIMP-1 expression in advanced stages of thyroid carcinoma prompted us to ask whether increased

 © Cancer Research Campaign 1999

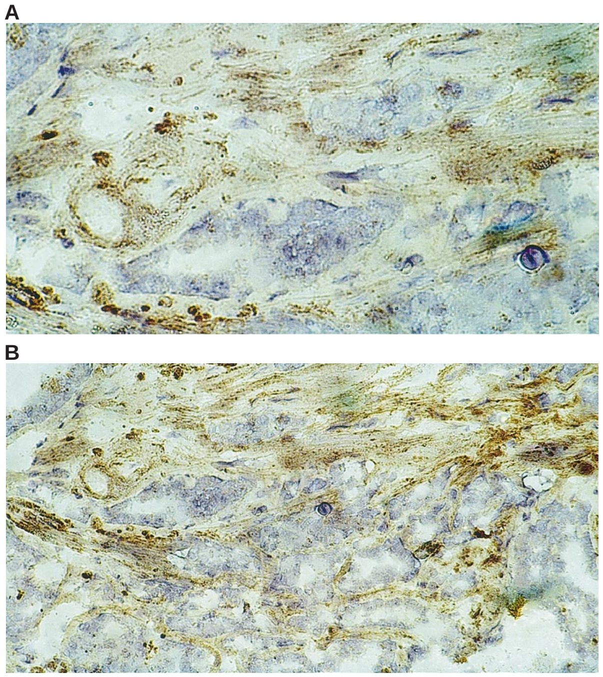

**Figure 3** Localization of TIMP-1 protein in an invasive thyroid papillary carcinoma specimen by immunohistochemistry. Section was stained with mAb against TIMP-1 using ABC immunoperoxidase staining system from Oncogene Science Inc. Note the strong TIMP-1 immunoreactivity within the stroma between the infiltrative tumour nests (magnification: (**A**) × 400; (**B**) × 200)

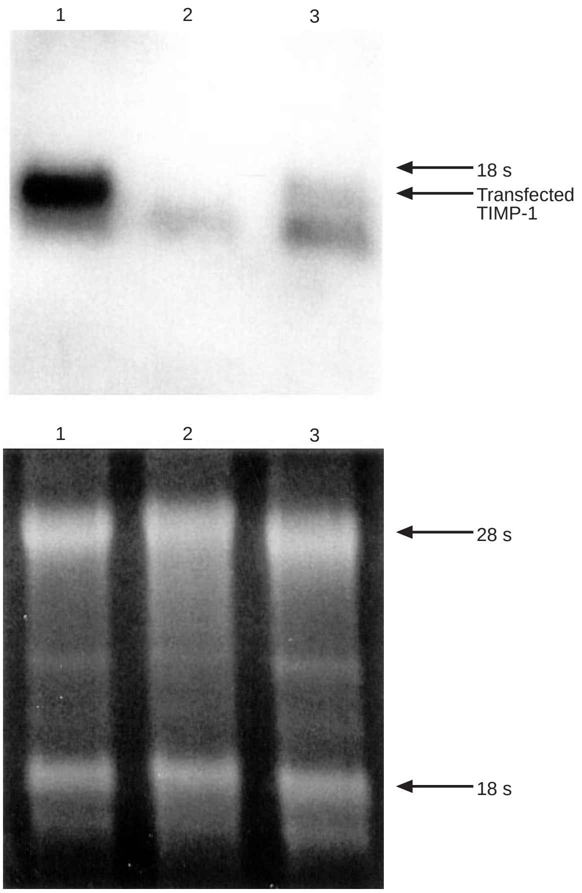

**Figure 4** TIMP-1 expression in transfected NPA thyroid carcinoma cell clones. Northern blot hybridization of full-length human TIMP-1 cDNA probe to 20 μg of total RNA extracted from human TIMP-1 transfected clones (upper panel). The endogenous TIMP-1 transcript is 0.9 kb and the transfected one is 1.1 kb which is indicated by an arrow. Lanes 1–3 represent high TIMP-1 expression clone (TP-1), vector alone transfected clone (control), and low TIMP-1 expression clone (TP-5), respectively. The actual RNA loading was monitored by ethidium bromide staining of RNA loaded for Northern blot analysis (lower panel)

TIMP-1 expression plays a role in counteracting the invasive behaviour of thyroid carcinoma. Gene transfer was used to stably express human TIMP-1 cDNA in a papillary thyroid carcinoma cell line, NPA. Two clones were selected for use in the in vitro invasion assay: one expressing high (TP-1), and the other low (TP-5) levels of exogenous TIMP-1 transcripts (Figure 4). As shown in Figure 5, high TIMP-1 expression decreased the invasiveness of NPA cells. As compared to the control, the invasive potential of TP-1 and TP-5 cells was reduced by 53% ($t$ = 7.48, $P$ < 0.0001) and 35% ($t$ = 4.86, $P$ < 0.001), respectively. Therefore, higher TIMP-1 expression was associated with increased inhibition of the invasive potential of NPA cells (TP-1 vs TP-5, $t$ = 2.23, $P$ < 0.05).

### DISCUSSION

The data presented herein show that TIMP-1 is expressed in both benign and malignant thyroid tumours, and that higher TIMP-1 expression is associated with advanced disease stages (stages III and IV). The findings seem to be contradictory to the proposed role of TIMP-1 as a tumour metastasis suppressor: if TIMP-1 acts as a functional inhibitor of tumour invasion and metastasis, its expression should inversely correlate with the aggressiveness of thyroid tumours. Indeed, such an inverse association has been documented in several (Albini et al, 1991; DeClerck et al 1991, 1992; Ponto et al, 1991; Khoka et al, 1992, 1994; Montgomery et al, 1994) but not all (Kussakowska et al, 1991; Lu et al, 1991; Visscher et al, 1994*a*, 1994*b*; Nakana et al, 1995; Zeng et al, 1995; Grignon et al, 1996) human and mouse tumours. One possible explanation for increased TIMP-1 expression in advanced stages of thyroid carcinoma is that it may represent a secondary event, i.e. invasive tumour cells may produce MMPs, which may induce surrounding stroma cells to produce TIMP-1 to contain tumour cells that would be otherwise even more invasive. Although the number of TIMP-1 transcripts is elevated, it may not be effective in both quantity or possibly quality (enzymatic activity) in counteracting MMPs produced by thyroid tumour cells. The high level of TIMP-1 may, therefore, reflect stroma response to elevated production of MMPs, and the imbalance between TIMP-1 and MMPs activities would result in ECM destruction and tumour invasion. A recent study by Soloway et al (1998) using targeted mutagenesis of TIMP-1 further revealed that tumour invasion and metastasis were influenced by TIMP-1 of the tumour and not of the host. Thus, it is likely that TIMP-1 level within tumour cells rather than in stroma cells determines their invasive behaviour.

The strong TIMP-1 immunostaining in stroma cells suggests that the high levels of TIMP-1 transcripts in advanced stages of thyroid carcinoma would likely represent a stroma response to tumour cell invasion. This is supported by the low TIMP-1 expression in both NPA and SW579 thyroid cancer cell lines that are derived from poorly differentiated thyroid carcinomas without stroma contamination. Several studies have demonstrated that

*© Cancer Research Campaign 1999* 

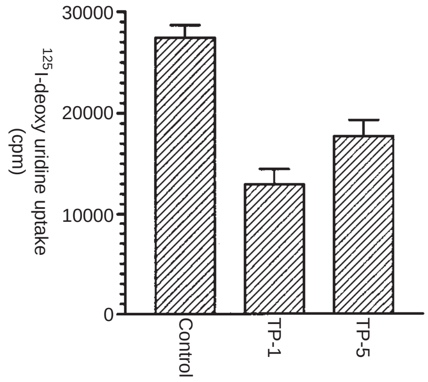

**Figure 5** Effect of high TIMP-1 expression on the invasive potential of NPA thyroid carcinoma cells. An in vitro invasion assay was carried out to compare and quantitate the invasiveness of NPA cells transfected with vector alone (control), NPA cells with high TIMP-1 expression (TP-1), and NPA cells with low TIMP-1 expression (TP-5). The cells were labelled with 1 μCi/ml $^{125}$I-deoxyuridine and placed in the upper compartment of a Transwell chamber for 96 h. The tumour cells penetrating into the lower surface of Matrigel®-coated filters were recovered with trypsin–EDTA and counted in a gamma counter. The experiment was performed in triplicate and repeated three times. Values are expressed as a mean ± SEM of three experiments increased TIMP-1 or TIMP-2 expression in advanced stages of malignancy derives predominantly from stroma cells rather than from cancer cells themselves (Kossakowska et al, 1991; Visscher et al, 1994*b*; Zeng et al, 1995; Grignon et al, 1996). In the study of elevated TIMP-1 mRNA levels in non-Hodgkin's lymphoma, Kossakowska et al (1991) showed by in situ hybridization that TIMP-1 transcripts were not localized to the malignant lymphocytes, but instead were present within stromal cells which account for only 20% of the total cells in each sample. Similar results were observed by Zeng et al (1995) in colorectal cancer. These investigators found that TIMP-1 mRNA was present predominantly in tumour stroma within spindle fibroblast-like cells. In invasive bladder and breast cancers, elevated TIMP-2 was mostly localized by immunohistochemistry in the tumour stroma around nests of invasive tumour cells (Visscher et al, 1994*b*; Grignon et al, 1996).

In order to determine whether TIMP-1 suppresses thyroid tumour cell invasion through basement membranes, we transfected NPA cells with human TIMP-1 cDNA and isolated clones with high and low TIMP-1 expression. Their invasive potentials were assessed by an in vitro tumour invasion assay in which reconstituted basement membranes (Matrigel®) were used. One major advantage of the system is that it allows quantitative analysis of the invasive activity of tumour cells, even though Matrigel® lacks the structural organization of the intact basement membranes (Albini et al, 1987; Hendrix et al, 1987). The assay measures the capability of a given tumour cell to attach to, degrade, and finally pass through, the matrix. These events are considered to be important steps in tumour cell invasion through basement membranes in vivo. In the present study, we demonstrated that NPA cells with high TIMP-1 expression were less invasive than those with low TIMP-1 level. The data suggest that TIMP-1 may indeed play an inhibitory role in thyroid tumour invasion and metastasis. Whether the expression of TIMP-1 from tumour vs stroma cells makes a difference in this regard is not known and requires further studies.

In summary, we have found elevated TIMP-1 mRNA in advanced stages of thyroid carcinoma. The increased levels of TIMP-1 transcripts likely come from stroma cells as an attempt to counteract tumour invasion and metastasis. NPA cells expressing high TIMP-1 transcripts have significantly less invasive potential in vitro than those with low TIMP-1 expression, supporting the role of TIMP-1 as a metastasis suppressor.

### ACKNOWLEDGEMENTS

We would like to thank Dr JA Fagin for providing NPA cell line and Ms R Khan for technical assistance in immunohistochemistry.

### REFERENCES

Albini A, Iwamoto Y, Kleinman HK, Martin GR, Aaronson SA, Kozlowski JM and McEwan RN (1987) A rapid in vitro assay for quantitating the invasive potential of tumor cells. *Cancer Res* **47**: 3239–3245

Albini A, Melchiori A, Santi L, Liotta LA, Brown PD and Stetler-Stevenson WG (1991) Tumor cell invasion inhibited by TIMP-2. *J Natl Cancer Inst* **83**: 775–779

Bernhard E, Gruber S and Muschel R (1994) Direct evidence linking MMP-9 (92 kDa gelatinase/collagenase) expression to the metastatic phenotype in transformed rat embryo cells. *Proc Natl Acad Sci USA* **91**: 4293–4297

Carmichael DF, Sommer A, Thompson RC, Anderson DC, Smith CG, Welgus HG and Stricklin GP (1986) Primary structure and cDNA cloning of human fibroblast collagenase inhibitor. *Proc Natl Acad Sci USA* **83**: 2407–2411

Chen C and Okayama H (1987) High-efficiency transformation of mammalian cells by plasmid DNA. *Mol Cell Biol* **7**: 2745–2752

Chomczynski P and Sacchi N (1987) Single-step method of RNA isolation by acid guanidinium thiocyanate–phenol–chloroform extraction. *Anal Biochem* **162**: 156–159

DeClerck YA, Yean T-D, Ratzkin BJ, Lu HS and Langley KE (1989) Purification and characterization of two related but distinct metalloproteinase inhibitors secreted by bovine aortic endothelial cells. *J Biol Chem* **264**: 17445–17453

DeClerck YA, Yean TD, Chan D, Shimada H and Langley KE (1991) Inhibition of tumor invasion of smooth muscle cell layers by recombinant human metalloproteinase inhibitor. *Cancer Res* **51**: 2151–2157

DeClerck YA, Perez N, Shimada H, Boone TC, Langley KE and Taylor SM Inhibition of invasion and metastasis in cells transfected with an inhibitor of metalloproteinases. *Cancer Res* **52**: 701–708

Goldberg GI, Marmer BL, Grant GA, Eisen AZ, Wilhelm SM and He C (1989) Human 72-kilodalton type IV collagenase forms a complex with tissue inhibitor of metalloproteinase designated TIMP-2. *Proc Natl Acad Sci USA* **86**: 8207–8211

Grignon D, Sakr W, Toth M, Ravery V, Angulo J, Shamsa F, Pontes JE, Crissman JC and Fridman R (1996) High levels of tissue inhibitor of metalloproteinase-2 (TIMP-2) expression are associated with poor outcome in invasive bladder cancer. *Cancer Res* **56**: 1654–1659

Hendrix MJ, Seftor EA, Seftor RE and Fidler IJ (1987) A simple quantitative assay for studying the invasive potential of high and low human metastatic variants. *Cancer Lett* **38**: 137–147

Hermanek P and Sobin LH (eds) (1987) *TNM Classification of Malignant Tumors*, pp. 33–35. Springer-Verlag: Berlin

Khokha R, Zimmer MJ, Wilson SM and Chambers AF (1992) Up-regulation of TIMP-1 expression in B16-F10 melanoma cells suppresses their metastatic ability in chick embryo. *Clin Exp Metastasis* **10**: 365–370

Khokha R (1994) Suppression of the tumorigenic and metastatic abilities of murine B16-F10 melanoma cells in vivo by the overexpression of the tissue inhibitor of the metalloproteinases-1. *J Natl Cancer Inst* **86**: 299–304

Kohn EC and Liotta LA (1995) Molecular insights into cancer invasion: strategies for prevention and intervention. *Cancer Res* **55**: 1856–1862

Kossakowska AE, Urbanski SJ and Edwards DR (1991) Tissue inhibitor of metalloproteinases-1 (TIMP-1) RNA is expressed at elevated levels in malignant non-Hodgkin's lymphomas. *Blood* **77**: 2475–2481

Leco KJ, Apte SS, Taniguchi GT, Hawkes SP, Khokha R, Schultz GA and Edwards DR (1997) Murine tissue inhibitor of metalloproteinases-4 (TIMP-4): cDNA isolation and expression in adult mouse tissues. *FEBS Lett* **401**: 213–217

Liotta LA and Stetler-Stevenson WG (1991) Tumor invasion and metastasis: an imbalance of positive and negative regulation. *Cancer Res* **51** (suppl.): 5054s–5059s

Liotta LA, Tryggvason K, Garbisa S, Hart I, Foltz CM and Shafie S (1980) Metastatic potential correlates with enzymatic degradation of basement membrane collagen. *Nature (Lond)* **284**: 67–68

© Cancer Research Campaign 1999

Liotta LA, Rao CN and Wewer UM (1986) Biochemical interactions of tumor cells with the basement membrane. *Annu Rev Biochem* **55**: 1037–1057

Liotta LA, Steeg PA and Stetler-Stevenson WG (1991) Cancer metastasis and angiogenesis: an imbalance of positive and negative regulation. *Cell* **64**: 327–336

Lu X, Levy M, Weinstein BI and Santella RM (1991) Immunological quantitation of levels of tissue inhibitor of metalloproteinase-1 in human colon cancer. *Cancer Res* **51**: 6231–6235

Matrisian LM Metalloproteinases and their inhibitors in tissue remodeling. *Trends Genet* **6**: 121–125

Montgomery AM, Mueller BM, Reisfeld RA, Taylor SM and Declerck YA (1994) Effect of tissue inhibitor of metalloproteinase-2 expression on the growth and spontaneous metastasis of a human melanoma cell line. *Cancer Res* **54**: 5467–5473

Murphy G, Reynolds J and Hembry RM (1989) Metalloproteinases and cancer invasion and metastasis. *Int J Cancer* **44**: 757–760

Nakano A, Tani E, Miyazaki K, Yamamoto Y and Furuyama J (1995) Matrix metalloproteinases and tissue inhibitors of metalloproteinases in human gliomas. *J Neurosurg* **83**: 298–307

Nakajima M, Welch DR, Belloni PN and Nicolson GL (1987) Degradation of basement membrane type IV collagen and lung subendothelial matrix by rat mammary adenocarcinoma cell clones of differing metastatic potentials. *Cancer Res* **47**: 4869–4876

Parhar RS, Shi Y, Zou MJ, Farid NR, Ernst P, Al-Sedairy ST (1995) Effects of cytokine-mediated modulation of NM23 expression on the invasion and metastatic behavior of B16F10 melanoma cells. *Int J Cancer* **60**: 204–210

Pavloff N, Staskus PW, Kishnani NS and Hawkes SP (1992) A new inhibitor of metalloproteinases from chicken: ChIMP-3. A third member of the TIMP family. *J Biol Chem* **267**: 17321–17326

Ponto A, Coulombe B and Skup D (1991) Decreased expression of tissue inhibitor of metalloproteinases in metastatic tumor cell leading to increased levels of collagenolytic activity. *Cancer Res* **51**: 2138–2143

Shi Y, Zou M, Schmidt H, Juhasz F, Stensky V, Robb D and Farid NR (1991) High rates of ras codon 61 mutation in thyroid tumors in an iodide-deficient area. *Cancer Res* **51**: 2690–2693

Soloway PD, Alexander CM, Werb Z and Jaenisch R (1996) Targeted mutagenesis of TIMP-1 reveals that lung tumor invasion is influenced by TIMP-1 genotype of the tumor but not by that of the host. *Oncogene* **13**: 2307–2314

Sreenath T, Matrisan L, Stetler-Stevenson W, Gattoni-Celli S and Pozzatti R (1992) Expression of matrix metalloproteinase genes in transformed rat cell lines of high and low metastatic potential. *Cancer Res* **52**: 4942–4947

Stetler-Stevenson WG, Krutzsch HC and Liotta LA (1989) Tissue inhibitor of metalloproteinase (TIMP-2): a new member of the metalloproteinase inhibitor family. *J Biol Chem* **264**: 17374–17378

Stetler-Stevenson WG, Aznavoorian S and Liotta LA (1993) Tumor cell interactions with the extracellular matrix during invasion and metastasis. *Annu Rev Cell Biol* **4**: 541–573

Visscher DW, Hoyhtya M, Ottosen SK, Liang C-M, Sarkar FH, Crissman JD and Fridman R (1994*a*) Enhanced expression of tissue inhibitor of metalloproteinase-2 (TIMP-2) in the stroma of breast carcinomas correlates with tumor recurrence. *Int J Cancer* **59**: 339–344

Visscher DW, Hoyhtya M, Ottosen SK, Liang CM, Sarkar FH, Crissman JC and Fridman R (1994*b*) Enhanced expression of tissue inhibitor of metalloproteinase-2 (TIMP-2) in the stroma of breast carcinomas correlates with tumor recurrence. *Int J Cancer* **59**: 339–344

Woessner JF (1990) Matrix metalloproteinases and their inhibitors in connective tissue remodeling. *FASEB J* **5**: 2145–2154

Zeng Z-S, Cohen AM, Zhang Z-F, Stetler-Stevenson W and Guillem JG (1995) Elevated tissue inhibitor of metalloproteinase 1 RNA in colorectal cancer stroma correlates with lymph node and distant metastases. *Clin Cancer Res* **1**: 899–906

*© Cancer Research Campaign 1999*
